# Lithium-silicate sol–gel bioactive glass and the effect of lithium precursor on structure–property relationships

**DOI:** 10.1007/s10971-016-4097-x

**Published:** 2016-06-23

**Authors:** Anthony L. B. Maçon, Manon Jacquemin, Samuel J. Page, Siwei Li, Sergio Bertazzo, Molly M. Stevens, John V. Hanna, Julian R. Jones

**Affiliations:** 1 Department of Materials, Imperial College London, London, SW7 2AZ UK; 2 Institute of Biomedical Engineering, Imperial College London, London, SW7 2AZ UK; 3 Department of Bioengineering, Imperial College London, London, SW7 2AZ UK; 4 Department of Physics, University of Warwick, Coventry, CV4 7AL UK

**Keywords:** Bioactive glass, Lithium, Sol–gel

## Abstract

**Abstract:**

This work reports the synthesis of lithium-silicate glass, containing 10 mol% of Li$_2$O by the sol–gel process, intended for the regeneration of cartilage. Lithium citrate and lithium nitrate were selected as lithium precursors. The effects of the lithium precursor on the sol–gel process, and the resulting glass structure, morphology, dissolution behaviour, chondrocyte viability and proliferation, were investigated. When lithium citrate was used, mesoporous glass containing lithium as a network modifier was obtained, whereas the use of lithium nitrate produced relatively dense glass-ceramic with the presence of lithium metasilicate, as shown by X-ray diffraction, $^{29}$Si and $^7$Li MAS NMR and nitrogen sorption data. Nitrate has a better affinity for lithium than citrate, leading to heterogeneous crystallisation from the mesopores, where lithium salts precipitated during drying. Citrate decomposed at a lower temperature, where the crystallisation of lithium-silicate crystal is not thermodynamically favourable. Upon decomposition of the citrate, a solid-state salt metathesis reaction between citrate and silanol occurred, followed by the diffusion of lithium within the structure of the glass. Both glass and glass-ceramic released silica and lithium ions in culture media, but release rate was lower for the glass-ceramic. Both samples did not affect chondrocyte viability and proliferation.

**Graphical Abstract:**

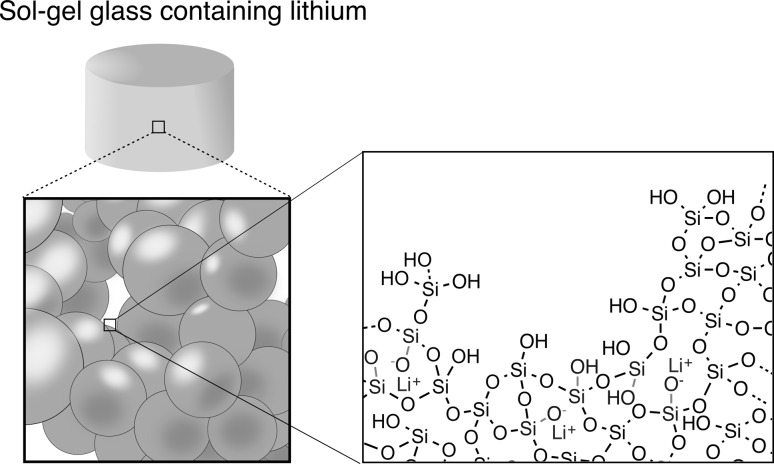

## Introduction

Lithium has been used clinically for more than half a century as a mood stabilising oral drug [1]. However, recent investigations have demonstrated that lithium can be used in other fields of medicine, especially for the regeneration of damaged bone and osteochondral tissue [2–7]. Arioka et al. showed that lithium chloride inhibits GSK-3, one of the main regulators of the Wnt/$\beta$-catenin pathway, which plays a crucial role in the differentiation of osteoblasts. In addition, Hui et al. [2] demonstrated that lithium can reduce the degradation of collagen via a cytokine-induced pro-inflammatory response in cartilage and Eslaminejad et al. [8] showed that lithium enhances the formation of proteoglycan-rich extracellular matrix in chondrogenic culture of mesenchymal stem cells. These findings are of particular interest since cartilage is one of the most challenging tissues to regenerate, since it is avascular.

There is great benefit in using glass to deliver active ions such as lithium because it can potentially provide sustained release, as release depends on dissolution rate of the glass. For release to be controlled, lithium must enter the silicate network. Khorami et al. substituted up to 12 wt% of sodium for lithium in 45S5 Bioglass^®^ (45 wt% SiO$_2,$ 24.5 wt% Na$_2$O, 24.5 wt% CaO and 6 wt% P$_2$O$_5$) [9]. Although lithium did not seem to alter the glass structure, a low lithium content (3 and 7 wt%) inhibited the formation of hydroxyapatite on the glass upon immersion in SBF. Similar compositions were made into porous scaffolds by Miguez-Pacheco et al. using foam replica techniques [10, 11]. Lithium has also been incorporated into ordered mesoporous sol–gel glass scaffolds (Li-MBG, 80 mol% SiO$_2,$ 10 mol% CaO, 5 mol% P$_2$O$_5$ and 5 mol% Li$_2$O) [12–14]. The release of lithium from Li-MBG had a beneficial effect on the proliferation and cementogenic differentiation of human periodontal ligament-derived cells via the activation of Wnt and SHH signalling pathways. In addition, Li-MBG enhanced the regeneration of osteochondral defects in rabbits compared to Li-free MBG.

Even though the data reported so far show that lithium can be incorporated into bioactive glass and be effective in regenerating damaged tissue, especially cartilage, the actual mechanism by which these glasses are performing is still unknown. In addition, recent studies reported that ions can act in pairs to generate combinational effects superior to the sum of the individual ionic contribution on cell metabolism [15–18]. For instance, when calcium ions and silica were both present in the culture of mouse pre-osteoblasts, an increase in the expression of osteocalcin was observed [15], which is a biomarker for bone formation. Thus, it is still unknown whether the outstanding performances of these glasses containing lithium ions are solely due to the release of lithium or whether lithium acts in combination with other ions already present in the physiological fluid or released from the materials. With regard to this potential combinatorial effect, it could be of great interest to investigate the release of lithium from less complex glass systems, by synthesising and testing a binary composition of lithium and silica.

Thus, the main aim of this work was to design such an investigation tool using the sol–gel process by conducting a structural study to see whether or not lithium can be incorporated as a network modifier in the amorphous silica network. The sol–gel synthesis of lithium-silicate glass-ceramic and ceramic has been reported in the literature using lithium nitrate and lithium alkoxide as precursors and by stabilising the gels at temperature favouring the crystallisation of lithium disilicate [19–22]. However, the synthesis and structural characterisation of an amorphous glass construct of lithium-silicate has never been reported. The objectives of this work were to produce amorphous SiO$_2$-Li$_2$O glass and to investigate the effect of the choice of lithium precursor on the structure and morphology of the glass. Once glasses containing lithium were obtained, their dissolution in immersion in cell culture was evaluated. The controlled delivery of lithium at therapeutic levels represents an exciting new strategy for cartilage repair, so the response of chondrocyte cells, which are responsible for cartilage production, to the new glasses was investigated.

## Results and discussion

### Effect of the lithium salt on the sol–gel process

Eslaminejad et al. [5, 8] studied the effect of Li$^+$ concentration on mesenchymal stem cells, from 1 to 10 mM. They found 5 mM to be optimal in terms of inducing chondrogenic differentiation of MSCs in a pellet culture system. Thus, 10 mol% LiO$_2$ was chosen for the glasses made here, as the glass produces dissolution products of 5 mM Li$^+$ in cell culture media when using a concentration of glass of 1.5 g L$^{-1}$ [23].

90 mol% SiO$_2$–10 mol% Li$_2$O binary glasses were synthesised using the sol–gel process by mixing lithium nitrate (90S10L(N)) or lithium citrate (90S10L(C)) with an acidic solution of hydrolysed tetraethylorthosilicate (TEOS) [24, 25]. A TEOS to water molar ratio of 12 was used in order to compare the structural data obtained here with previous reports on sol–gel bioactive glass structure evolution [26–29]. Pure silica gels were also prepared using similar protocols as a control (Li-free, 100S). Upon the addition of the lithium precursors, the pH of the solution containing lithium citrate increased from 1 to 5.3, whereas no change was observed with lithium nitrate or 100S. As a consequence, differences in gelation were noticed between the two solutions: (1) The sol containing lithium nitrate gelled in 3 days, which was expected as the condensation reaction is the limiting reaction in the sol–gel process when performed at a pH < 2 (isoelectric point of silicic acid pH = 2) [30]; (2) the sol containing lithium citrate gelled in 1 h. Upon solubilisation of lithium citrate in the sol, lithium dissociated from its counter ion. This release of citric acid (weak acid, pK$_{a1}$ = 3.13, pK$_{a2}$ = 4.76 and pK$_{a3}$ = 6.40) caused an increase in pH above the isoelectric point of silicic acid, which subsequently changed the kinetics of condensation of the silica network [31]. Aliquots of the pore liquor (initial solvent + by-products of the hydrolysis/condensation reaction), after ageing, were analysed by inductively coupled plasma (ICP). With both precursors, the concentration of lithium ions were found to be statistically equivalent at 0.12 mol L$^{-1},$ meaning that lithium was entirely solubilised in the pore liquor and not incorporated in the silica network. This result was similar to the observation of Ca incorporation made by Lin et al. for the sol–gel synthesis of the binary CaO–SiO$_2$ silica glass using Ca(NO$_3$)$_2\cdot$4H$_2$O [26, 29].

### Crystallisation behaviour upon thermal stabilisation

**Fig. 1:**
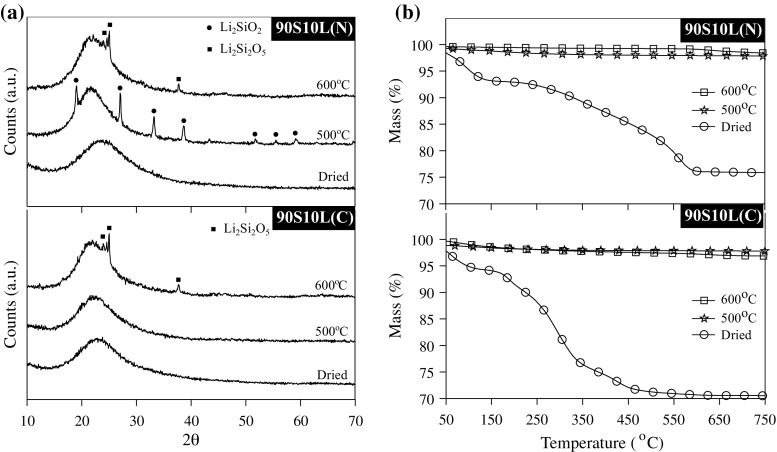
**a** XRD patterns and **b** TGA traces recorded before and after thermal stabilisation at 500 and 600 °C of the sol–gel glasses made with 10 % lithium nitrate (90S10L(N)) or 10 % lithium citrate (90S10L(N))

Upon thermal stabilisation, sol–gel-derived glasses can crystallise and the devitrification process can be greatly affected by the synthesis parameters and the gel composition [21, 22, 32, 33]. Thus, it is important to carefully select the stabilisation parameters in order to yield a glass network that contains lithium. Stabilisation was conducted at increments of 50 from 400 °C up to 600 °C and monitored using XRD and TGA (Fig. 1). Regardless of the composition and the temperature, a broad amorphous halo, centred around 24° 2$\theta,$ was detected by XRD, which did not diminish in counts when lithium and silica crystallised. Significant difference was observed between 90S10L(N) and 90S10L(C) in terms of crystallisation. Gel prepared with lithium nitrate formed lithium metasilicate when heated to 450 °C. On reaching 600 °C, lithium disilicate was exclusively detected. Samples prepared with lithium citrate did not crystallise until 550 °C, forming lithium disilicate. After stabilisation, TGA was conducted to evaluate whether the lithium counter anions (e.g. citrate or nitrate) were fully decomposed/desorbed. The citrate fully decomposed at 400 °C, whereas a temperature of 500 °C was required for nitrate decomposition.

It has already been observed that, when nitrate is present during the synthesis of lithium-silicate gels, the temperature of crystallisation offset significantly decreased [22, 34]. In addition, a large excess of water, corresponding to a water to TEOS molar ratio above 15, also lowered the crystallisation temperature and favoured the formation of lithium metasilicate [34]. However, these reports described the sol–gel synthesis of lithium-silicate using an equimolar amount of silicon and lithium. No systematic study has been reported for lithium-silicate gels synthesised with high silica content as presented in this report. Schwartz et al. did not observe the formation of lithium metasilicate when targeting 15 mol% of Li$_2$O using lithium nitrate, with a water to TEOS molar ratio of 2 (as opposed to 12 here) and a single stage thermal stabilisation of up to 600 °C [19]. Likewise, Chen and James, targeting a 10 mol% Li$_2$O–90 mol% SiO$_2$ composition and using a similar water content as Schwartz et al., only observed the crystallisation of lithium disilicate at 650 °C, similar to melt-derived equivalent [20]. An increase in the water content therefore favours early crystallisation [20].

**Fig. 2:**
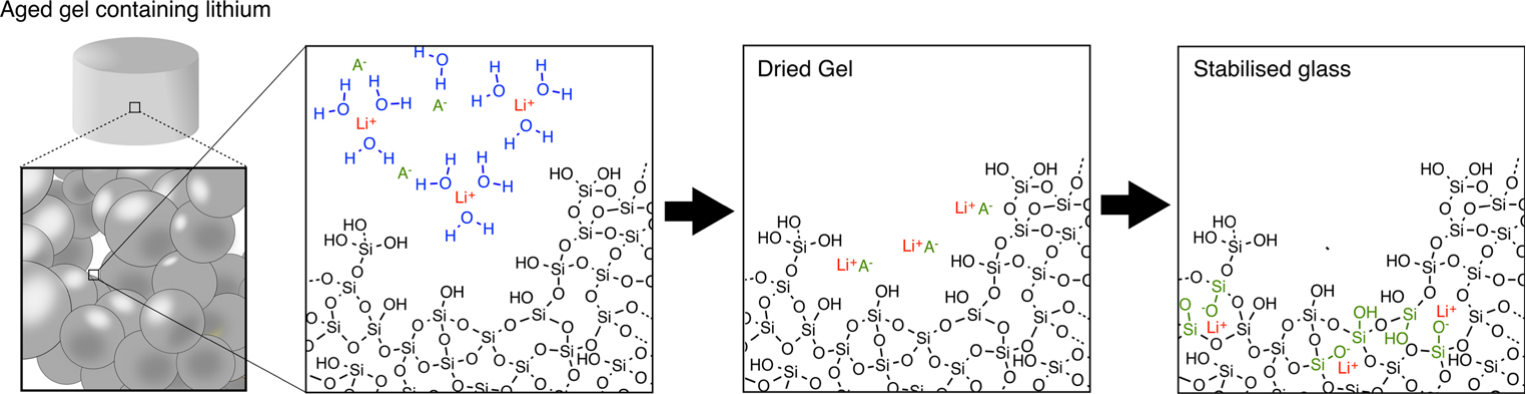
Schematic representing the precipitation of lithium salt within the porous structure of silica gels, subsequently followed by the diffusion of lithium, induced by thermal stabilisation, within the silicate networks, decreasing connectivity of the network

This implies that the difference in crystallisation of melt- and gel-derived glasses cannot be simply summarised by their difference in OH content or in surface area as suggested elsewhere [32]. The nature of salt/alkoxide precursor used as a network modifier must be taken into account to fully understand crystallisation events. In the present case, the following mechanism is hypothesised: Since the analysis of the pore liquor revealed that lithium is solvated in the pore liquor during ageing, we assume that the silanol residues (Si-OH) are poor lithium chelators at the pH at which the synthesis was carried out [35]. Thus, upon drying, lithium citrate or nitrate reprecipitated within the pores and on the surface of the silica gel (Fig. 2). Due to the low decomposition temperature of citrate and the relative low bonding strength between lithium and citrate [36], a solid-state salt metathesis reaction occurred between lithium citrate and the silanol residues at a temperature where the crystallisation of lithium and silica is not thermodynamically favourable [35], as (Eq. 1):

1 $$\begin{aligned} RCOOLi + SiOH \rightarrow SiOLi + RCOOH \end{aligned}$$

Lithium subsequently diffuses within the bulk silica network in a similar manner to calcium, as proposed by Lin et al. [26, 29]. In contrast, nitrate has a better affinity for lithium and decomposes at a higher temperature [37, 38]. Thus, we can assume that the higher concentration of lithium in the mesopores and the elevated temperature at the start of the metathesis reaction favourably induced the formation of a surface seed crystals (heterogeneous crystallisation), which grew during the second stage of the stabilisation at 500 °C for 5 h. This implies that the surface area and pore volume had a direct impact on the formation of this seed crystal, which could explain why higher water content accelerated crystallisation.

**Fig. 3:**
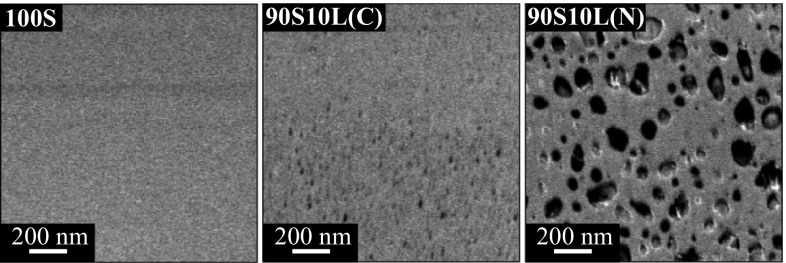
Backscattered SEM micrographs from a FIB cross section of 100S and lithium containing sol–gel silica glasses after stabilisation at 500 °C

The heterogenous crystallisation was indirectly verified by backscattered electron (BSE) imaging of the glass FIB cross section after stabilisation at 500 °C, as shown in Fig. 3. FIB cross-sectioning for structural investigations is of particular interest as the section does not show the weakest mechanical planes. 100S and 90S10L(C) had characteristics of a homogeneous amorphous material, whereas 90S10L(N) had two distinctive phases with a highly contrasted image. Lithium-silicate crystal domains are known to give very little backscattered signal due to the low atomic weight of lithium [39, 40]. Thus, the dark region represents lithium metasilicate and the remaining volume is expected to be silica. The spherical morphology of the crystalline domain is typical of the growth of a metastable phase from an heterogeneous mix [41].

To conclude on the thermal stabilisation study, a temperature of 500 °C was selected for stabilisation, as it is the lowest temperature where both lithium counter anions fully decomposed.

### Effect of crystallisation on the structure and morphology

The structure and morphology of 90S10L was studied by one pulse solid-state magic-angle spinning nuclear magnetic resonance (MAS NMR) and nitrogen sorption. Structural or morphological variations could result in variations in the degradation properties of the glasses [42]. In addition, differences in $^{29}$Si and $^7$Li MAS NMR signal upon stabilisation could confirm the diffusion/crystallisation mechanism made in the previous section.

**Table 1:** Summary of the structural and morphological characterisation carried out on the gels before and after stabilisation at 500 °C

Stabilisation	Lithium	Proportions (%)				D$_{Q}$	Surface area	Pore volume	Pore ∅
Temperature	Precursor	Q$^{1}$	Q$^{2}$	Q$^{3}$	Q$^{4}$	(%)	(m$^2$ g$^{-1}$)	(cm$^3$ g$^{-1}$)	(nm)
Dried	Nitrate	–	5.5	26.2	68.4	90.8	353	0.308	3.05
130 °C	Citrate	2.7	4.8	20.5	72.0	90.5	298	0.595	9.51
	100S	2.7	6.9	22.5	67.2	88.2	720	0.674	3.04
500 °C	Nitrate	2.2	6.0	14.6	77.2	91.7	35	0.659	5.65
	Citrate	0.1	14.8	20.5	64.5	87.3	389	0.798	9.75
	100S	2.1	5.3	21.8	70.8	90.3	692	0.697	3.81

D$_{Q}$ represents the degree of condensation of the Q species (e.g. connectivity). Q$^{n}$ are Si tetrahedral units with n the number of bridging oxygen bonds. Pore ∅ represents the pore diameter of the gels/glass/glass-ceramics (BJH method)

**Fig. 4:**
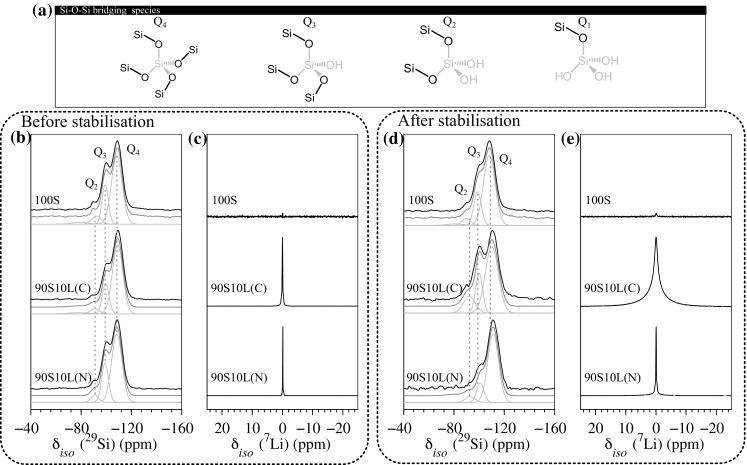
**a** Representation of the different silicate species that could be detected by $^{29}$Si NMR; $^{29}$Si MAS NMR spectra of 100S and 10 mol% lithium doped sol–gel glasses after drying; **b**, **c** before stabilisation and **d**, **e** after heat stabilisation at 500 °C

Thus, the connectivity of the silica networks, before and after stabilisation at 500 °C, was evaluated by MAS NMR [43, 44]. Deconvoluted $^{29}$Si MAS NMR spectra were used to quantify the number of bridging oxygen bonds, *n*, that a silicon atom can have with other surrounding silica tetrahedra as each Q$^n$ species can be detected at different chemical shifts: −72, −81, −91, −100 and −108 ppm, which correspond to Q$^0,$ Q$^1,$ Q$^2,$ Q$^3,$ and Q$^4,$ respectively. A structural representation of the Q species is shown in Fig. 4a. The proportion of each Q$^{n}$ species and the degree of condensation D$_Q,$ obtained from the deconvoluted spectra are summarised in Table 1. Figure 4-b shows the $^{29}$Si MAS NMR spectra of the gels before thermal stabilisation, which were all composed of a mixture of Q$^2$ to Q$^4$ species, representative of a condensation reaction occurring below pH 7 [30]. 90S10L(C) presented a higher fraction of Q$^4$ due to its higher pH of condensation as compared to 100S or when lithium nitrate was used. The signals given by lithium from 90S10L (N) or (C) were well defined with a sharp resonance centred around 0 ppm with a full width at maximum (FWHM) not exceeding 0.2 Hz (Fig. 4c). This is characteristic of lithium in a highly ordered structure, which confirms that lithium reprecipitated as salts within the open mesoporous structure of the silica gel [44].

Upon stabilisation, the connectivity of the silicate network changed and in different proportion depending of the sample as shown in Fig. 4d. The degree of condensation of 100S increased from 88.2 to 90.3 % which is the result of an increase in Q$^{4}$ to the detriment of the other Q species. This was due to the condensation of free silanols, catalysed by the heat [31]. 90S10L(N) also increased in connectivity with D$_Q$ rising from 90.8 to 91.7 %, which was due to the cross-linking of Q$^3$ species. This suggests that the crystallisation of Li$_2$SiO$_2$ induced solid-phase separation and the formation of a glass-ceramic. In addition, a slight increase in Q$^2$ was also observed and might be the result of the crystallisation of lithium with silica as lithium metasilicate which is a tectosilicate crystal (orthorhombic lattice), meaning that two oxygens of the silicon tetrahedron are charged balanced with cations [22, 45]. However, this statement should be taken with care as the variation is within the error of the measurements [26, 29]. The silicate connectivity of 90S10L(C) decreased after thermal stabilisation, with a decrease in the proportion of Q$^4$ species and an increase in the proportion of Q$^2$. This agrees with an increase of the FWHM of the lithium resonance from 0.2 to 6 Hz, suggesting that lithium was now residing in an amorphous network characterised by short range disorder. The change in connectivity suggests that lithium acted as a network modifier and diffused through the silica network by breaking existing bridging oxygen to charge balance the structure [28]. This reinforces the findings of Lin et al. who hypothesised this mechanism for Ca$^{2+}$ incorporation [26, 29]. In addition, the homogenous distribution of lithium within the structure can also explain why lithium disilicate formed, in the citrate samples, without any transient metastable phase, reinforcing the hypothesis of crystallisation from an heterogenous mix when lithium nitrate was used.

**Fig. 5:**
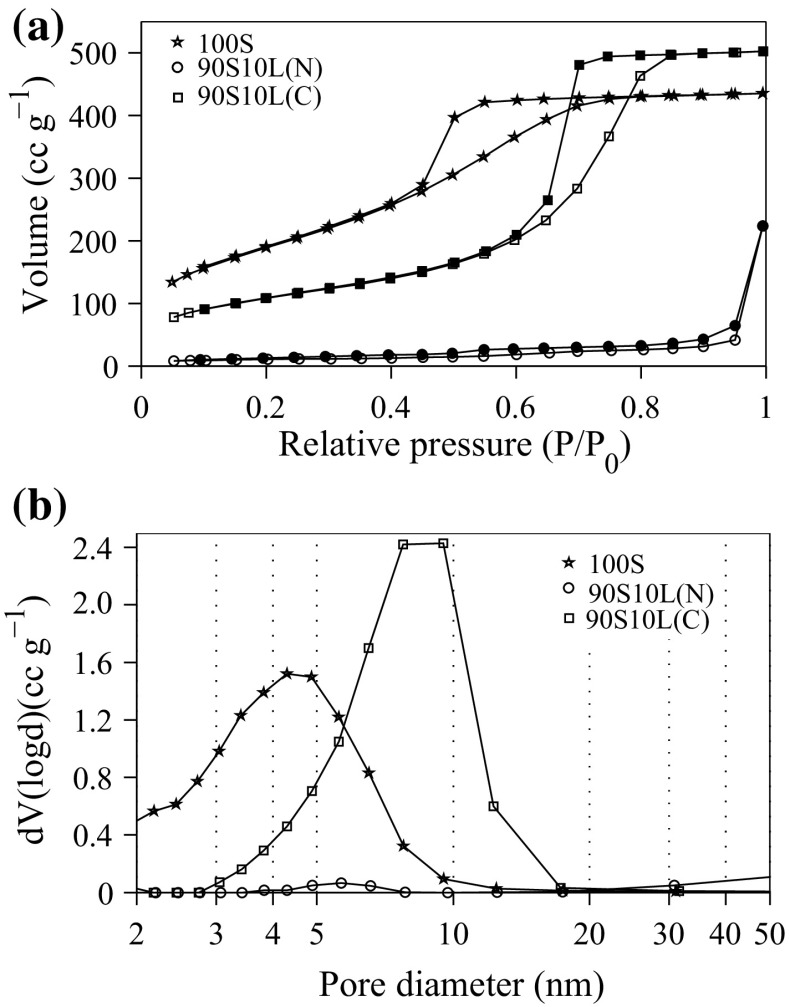
**a** Nitrogen sorption isotherms (77 K) after thermal stabilisation of the samples at 500 °C. **b** Pore size distributions obtained by applying the BJH algorithm on the desorption branches of the isotherms from **a**

The morphology of the samples was studied by analysing the nitrogen sorption isotherm (77 K). Figure 5 shows the isotherms and pore size distributions of the gel after heat stabilisation. All values obtained from BET and BJH algorithms are summarised in Table 1. A mix of Type III/IV isotherms with a Type A hysteresis was obtained from all the glass before stabilisation, meaning that the condensation of the nitrogen occurred in interconnected spherical/cylindrical mesopores [46]. The modal pore diameter of 100S and 90S10L(N) before stabilisation was approximatively equal to 3 nm. However, the surface area of 100S, 692 m$^2$ g$^{-1},$ was half that of 90S10L(N) at 353 m$^2$ g$^{-1}$. This is likely to be the result of the recrystallisation of lithium nitrate within the pores in 90S10L(N), which obstructed the flow of nitrogen within the gels. The pore volume of the 90S10(C) before stabilisation was higher than the other gels due to the higher pH at which the condensation took place. Upon stabilisation, the pore volume and surface area of 100S remained unchanged. The crystallisation of lithium metasilicate induced a significant decrease in surface area, reaching 35 m$^2$ g$^{-1}$. This reinforces the hypothesis that the heterogeneous crystallisation occurred with 90S10L(N) where the crystal nuclei were located within, or close to, the pores of the gel. The diffusion and removal of the citrate caused an increase in the pore volume, inducing an increase in surface area from 298 to 387 m$^2$ g$^{-1}$ while retaining a constant pore diameter.

### Effect of the crystallisation on the in vitro performance

The effect of the crystallisation on the release of lithium and silica in Dulbecco’s modified Eagle’s medium (DMEM) and the effect on chondrocyte proliferation and attachment were investigated [23, 47, 48].

**Fig. 6:**
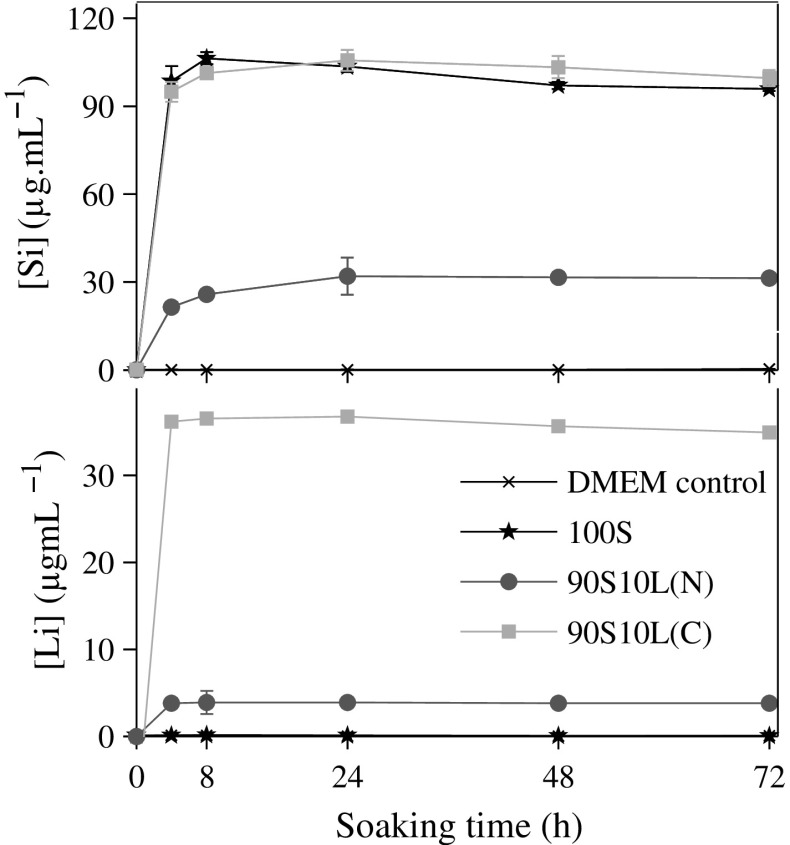
Lithium (Li) and silicon (Si) concentration profiles upon immersion of 100S and 10 mol% Li$_2$O sol–gel glass/glass-ceramic stabilised at 500 °C in D-MEM culture medium over 3 days. *Error bars* represent the standard deviation, $n=3$

Upon immersion of 90S10L(C) and 90S10L(N) in DMEM without cells, lithium and silica were successfully released. Substantial differences in concentrations between the samples were measured after 3 days of immersion (Fig. 6). The silicon release profile of 90S10L(C) followed closely that of 100S, with a rapid increase in concentration within the first 8 h, reaching 99.6 $\upmu$g mL$^{-1}$ (3.6 mM), which stayed constant thereafter. Burst release of lithium was observed from 90S10L(C) within the first 4 h of immersion, reaching 34.9 $\upmu$g mL$^{-1}$ (5.02 mM), amounting to 95 % of the lithium present initially in the glass. Slight increase in the pH was observed, from 7.40 to 7.55. These observations were similar to those made with amorphous binary silica-calcium sol–gel glass (80 mol% SiO$_2$–20 mol% CaO) when immersed in SBF [25]. Thus, it is likely that 90S10L(C) followed the same dissolution mechanism with an exchange of the lithium from the glass with the surrounding H$_3$O$^+$ from the media, causing the pH to increase [23].

Silicon from 90S10L(N) was released at a slower rate than 90S10Li(C), reaching a steady concentration of 31.3 $\upmu$g mL$^{-1}$ (1.1 mM) after 24 h of immersion. The reduction in dissolution behaviour is likely to be a combinational effect of the decrease in specific surface area, the higher network connectivity and the crystallisation of lithium metasilicate. The dissolution reaction of lithium metasilicate has a positive free energy of $\approx$12 kcal mol$^{-1}$ from 25 to 90 °C at pH 7, meaning that under the immersion conditions used here the dissolution of the crystalline phase is not spontaneous and not favourable [49]. Thus, it is likely that the lithium released in solution, 3.8 $\upmu$g mL$^{-1}$ (0.5 mM) at 72 h, came from the fraction that had not crystallised yet, or entire crystals were lost to the DMEM.

The level of silica from 100S and its release rate were both higher than previously reported, at any given time point [25, 50, 51]. It is likely to be due to the lower stabilisation temperature used here. Above 600 °C, 100S undergoes extensive condensation of the Si-OH located at surface of the pores leaving the silica network exclusively composed of Q$^{4}$ species, whereas at 500 °C (used here) a mixture of Q$^{2},$ Q$^{3}$ and Q$^{4}$ species was obtained [26, 29, 30]. When 100S was stabilised above 600 °C, an activation energy barrier of 14–24 kcal mol$^{-1}$ had to be crossed for the hydroxylation of the Q$^4$ species to occur prior to the release of silica, which in turn slowed down the dissolution [52–54].

**Fig. 7:**
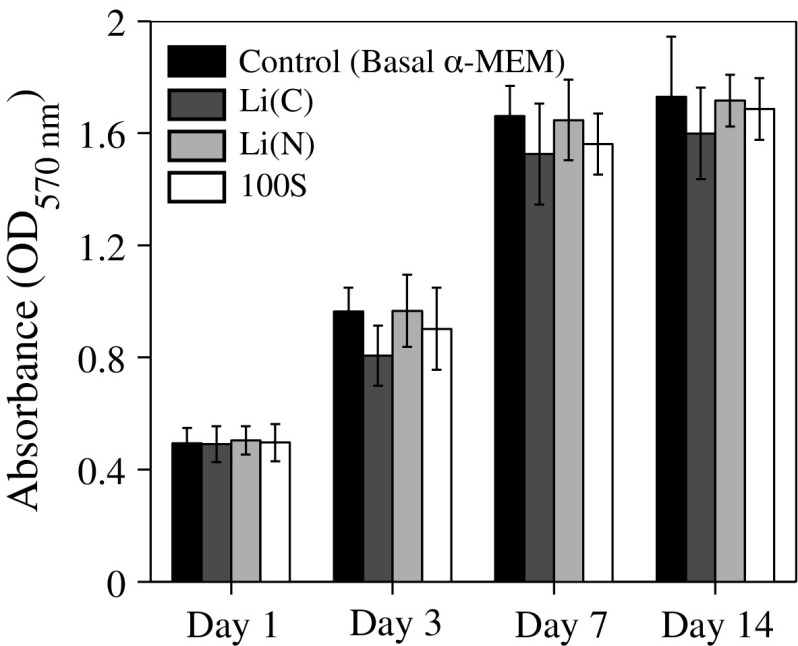
Cell viability test of ATDC5 chondrocytes exposed to the dissolution products of 100S and SiO$_2$–10 mol% Li$_2$O sol–gel glass/glass-ceramic stabilised at 500 °C

**Fig. 8:**
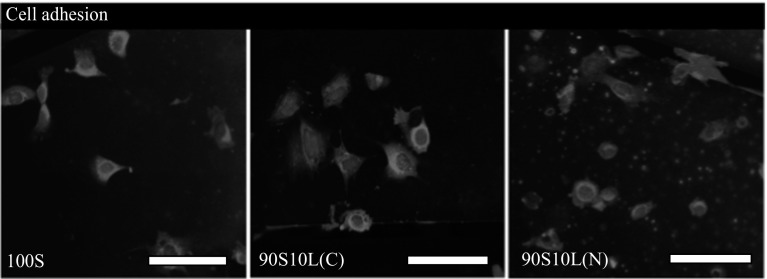
Confocal images of ATDC5 chondrocytes seeded on the glasses and cultured for 24 h with F-actin filaments in *red*, DNA in *blue* and vimentin in *green*. *Scale bar* 50 $\upmu$m (Color figure online)

Cell viability and the ability to support cell attachment and growth are two of the key criteria to consider for biological applications of a material. Cell viability was assessed by measuring the metabolic activity of ATDC5 cells cultured in the presence of glass dissolution products. The MTT assay confirmed that the 100S and both types of 90S10L glasses/glass-ceramic were not toxic to the cells at the tested concentration (Fig. 7). Cells were capable of continuous growth over a period of 14 days in the presence of dissolution products of the three sol–gel-derived glass reported in the present study (Fig. 7). There was no significant difference compared to basal DMEM control. Cell attachment on the sol–gel glass was examined by immunohistochemical staining and confocal microscopy. Following 3 day of culture, ATDC5 cells adhered to all three types of sol–gel glass ( Fig. 8). Functional viable cell attachment was evidenced by the expression of two of the major cytoskeleton proteins Vimentin, intermediate filament, and F-actin, microfilament. These findings suggest that lithium-silicate sol–gel-derived bioactive glasses are not cytotoxic and possess the suitable surface properties to support functional cell attachment and growth. Future studies will therefore focus on the effect of 90S10L glasses/glass-ceramic and their dissolution products on chondrogenic differentiation and cartilaginous matrix formation.

## Conclusions

In this work, we have shown that the precursor selection for the design of bioactive glasses affects the sol–gel process and the structure and properties of the final material. Citrate salts have a lower decomposition temperature than nitrate, which proved to an advantage as gels prepared from lithium citrate produced glasses with lithium within the silicate structure as a network modifier. Nitrate could not be decomposed without the formation of lithium metasilicate. This impaired the degradation behaviour of the glass without affecting the activity of chondrocyte cells on the sample. This glass or glass-ceramic could used as a tool for further investigation of the cumulative effect of lithium and silica on chondrocyte genetic pathways involved in the regeneration of damaged cartilage tissues.

## Experimental procedures

### Materials

All reagents were purchased from Sigma-Aldrich UK and used as received.

### SiO$_2$–Li$_2$O sol–gel glass synthesis

The protocol used for the glass synthesis was adapted from Saravanapavan and Hench [25]. Tetraethylorthosilicate (TEOS) was hydrolysed first, for 1 h, in presence of deionised water (DiW) and nitric acid (2 M) by vigorous agitation in a PTFE mould. The molar ratio of TEOS to DiW was fixed to 1:12, whereas nitric acid (2 M) was added by volume amounting to 1/6 of the volume of DiW. Then, the lithium precursor (lithium nitrate or lithium citrate tribasic tetrahydrate) was slowly added to the sol with moderate agitation, targeting an molar ratio of oxide equal to SiO$_2$:Li$_2$O = 90:10. Glass without lithium was also synthesised as a control. After 1 h, the moulds were tightly sealed and left for 3 days at room temperature for the sol to gel and subsequently aged at 60 °C for 3 days. The glasses were then dried up to 130 °C using a 3-stage programme (60 °C for 20 h, 90 °C for 24 h and 130 °C for 40 h, ramp = 1 °C min$^{-1}$) and stabilised to the temperature of interest through a 2-stage programme (300 °C for 2 h, temperature of interest for 5 h, ramp $=$ 1 °C min$^{-1}$). The stabilised glasses were then ground and sieved (100 $\upmu$m mesh size). Discs (0.5 mm diameter) were also made following the same protocols to assess chondrocyte adhesion.

### Characterisation

#### X-ray powder diffraction (XRD)

Analysis was performed with a Bruker D2 desktop XRD from 10° to 70° 2$\theta ,$ using a 0.02 step size for 15 min without spinning. The radiation source was a Ni filtered Cu$\kappa _{\alpha }$. Samples were place on an amorphous silicon disc for measurement.

#### Thermogravimetry analysis (TGA)

 TGA was performed using a Netzsch sta 449 c in air. The sample was placed in a platinum crucible and heated up to 1000 °C at 10 °C min$^{-1}$.

#### Nitrogen sorption

Samples were degassed (Degasser, Quantachrome P < 1 mbar) for 12 h prior to measurement. In addition, a heating jacket, at 150 °C, was used and placed around the working capillary during degassing. Nitrogen sorption was carried out on a Quantachrome Autosorb AS6 multi-station with 20 absorption and 20 desorption points. Specific surface area was obtained from the 5 first points of the absorption branch of the isotherm (P/P$_0$ < 0.3) using the BET equation [55], while the pore distribution, pore diameter, and pore volume were obtained using the BJH method [56] on the desorption branch.

#### SEM analyses and focused ion beam

Samples were secured to an aluminium sample holder with carbon tape and carbon paste, which was then coated with 10 nm gold in a sputter coater (Quorum Technologies Sputter Coater model K575X). Following the coating procedure, samples were imaged by SEM (Carl Zeiss - Auriga) operating at 5 kV with a gallium ion beam operated at 30 kV. The samples was sectioned using 4-nA gallium current. The region exposed to milling was polished with 50-pA current and imaged by a backscattering detector with the electron beam operating at 1.5 V.

#### Magic angle spinning–solid state nuclear magnetic resonance (MAS NMR) spectroscopy

$^7$Li and $^{29}$Si single pulse MAS NMR measurements were performed at 7.0 T using a Varian/Chemagnetics Infinity Plus spectrometer operating at Larmor frequencies of 121. 48 and 69.62 MHz, respectively. The $^7$Li experiments were performed using a Bruker 4 mm HX probe which enabled a MAS frequency of 10 kHz to be implemented. Flip angle calibration was performed on a 9.7 M LiCl solution from which a ‘non-selective’ (solution) $\pi$/2 pulse time of 4 $\upmu$s was measured. This corresponds to a ‘selective’ (solid) pulse time of 2 $\upmu$s. All measurements were undertaken with a $\pi$/2 tip angle along with a recycle delay between excitation pulses of 10 s. All $^7$Li centre-of-gravity (apparent) shifts were reported against the IUPAC-recommended primary reference of LiCl (7 M in D$_2$O, $\delta$ 0.0 ppm) [57]. The $^{29}$Si experiments were performed using a Bruker 7 mm HX probe which enabled a MAS frequency of 5 kHz to be implemented. Flip angle calibration was performed on kaolinite from which a $\pi$/2 pulse time of 5.5 $\upmu$s was measured. All measurements were undertaken with a $\pi$/2 tip angle along with a recycle delay between subsequent pulses of 240 s. All $^{29}$Si isotropic chemical shifts were reported against the IUPAC-recommended primary reference of Me$_4$Si (1 % in CDCl$_3,$ $\delta$ 0.0 ppm), via a solid kaolinite secondary reference from which the resonance exhibits a known shift of $-92.0$ ppm [57].

#### Dissolution test

Glasses were immersed in Dulbecco’s modified Eagle’s medium (DMEM) supplemented with penicillin streptomycin (1 v/v%) and bovine serum (10 v/v%) at a fixed glass to media ratio of 1.5 mg mL$^{-1}$ [23]. The glass and the media (100 mL) were placed in an airtight polyethylene container and subsequently placed in a orbital shaker at 37 °C rotating at 120 r.p.m. 1 mL aliquots were taken at 4, 8, 24, 48 and 72 h in order to measure the pH and evaluate the ionic concentration profiles of the media. The concentration of silicon, lithium, calcium and phosphorus were determined using a Thermo Scientific iCaP 6300 Duo inductively coupled plasma–optical emission spectrometer (ICP–OES). Sample solutions were prepared by diluting the collected aliquots with 2 M HNO$_3$ by a factor of 10 and filtered using 0.45 $\upmu$m cellulose filters. Mixed standards of silicon, lithium, calcium and phosphorus were prepared at 0, 2, 5, 20 and 40 $\upmu$g mL$^{1}$ for calibration. Silicon, phosphorus and lithium were measured in the axial direction of the plasma flame, whereas calcium was measured in the radial direction as recommended by the software. All samples were run in triplicate for statistical analysis, and a DMEM alone was incubated under the same conditions and used as a control.

#### Cell viability and attachment

Chondrogenic cell line ATDC5 was culture expanded in DMEM supplemented with 100 unit mL$^{-1}$ penicillin, 100 $\upmu$g mL$^{-1}$ streptomycin, 5 % (v/v) FCS (foetal calf serum) and 1$\times$ ITS liquid supplement (10 $\upmu$g mL$^{-1}$ insulin, 5.5 $\upmu$g mL$^{-1}$ transferrin and 5 ng ml$^{-1}$ selenite premix). To determine the potential cytotoxicity effect of 90SiO$_2$-10Li$_2$O and 100S sol–gel glasses on ATDC5 cells, dissolution products released by the glass granules (1.5 $\upmu$g mL$^{-1}$) over a 3 day period at 37 °C were prepared. The dissolution products were filter sterilised and supplemented with 5 % (v/v) FCS and 1× ITS liquid supplement prior to use in cell viability assays. ATDC5 cells were passaged using 500 $\upmu$g mL$^{-1}$ trypsin-EDTA (ethylene diamine tetra-acetic acid) and seeded on 24-well plates at $5\times 10^{3}$ cells per cm$^2$ and left to grow in basal DMEM for 24 h. The culture media was then replaced with the dissolution products of each glass composition for further 1, 4, 7 and 14 days. At each time point, the culture media was removed and cells were incubated with MTT solution (3-(4,5-dimethylthiazol-2-yl)-2,5-diphenyltetrazolium bromide, 1 mg/ml in serum-free DMEM) for 3 h. The resulting formazan derivatives were dissolved with DMSO (dimethyl sulfoxide) for 5 minutes and the optical density was determined spectrophotometrically at 570 nm using a SpectraMax M5microplate reader. Cell viability in each glass composition was assayed in triplicate and, basal DMEM was used as positive control. For cell attachment studies, discs (5 mm diameter, 1 mm thick) of each glass composition were manufactured and sterilised with 70 % ethanol. Monolayer cultured ATDC5 cells were harvested and suspended in basal DMEM at a concentration $1\times 10^{6}$ cells mL$^{-1}$. 10 $\upmu$L of cell suspension was seeded onto each glass disc and incubated for 2 h. Each cell-seeded glass discs was then immersed in fresh basal DMEM and cultured for further 3 days before fixation with 4 % paraformaldehyde (PFA) for immunohistochemical labelling of Vimentin and F-actin. All samples were nuclei-stained with DAPI (0.1 $\upmu$g mL$^{-1}$ in PBS). Samples were imaged under confocal microscopy (Leica SP5 MP laser scanning confocal microscope and software, Leica Microsystems, Wetzlar, Germany).
